# Application of High Voltage Electrical Discharge Treatment to Improve Wheat Germination and Early Growth under Drought and Salinity Conditions

**DOI:** 10.3390/plants10102137

**Published:** 2021-10-09

**Authors:** Tihana Marček, Tihomir Kovač, Katarina Jukić, Ante Lončarić, Maja Ižaković

**Affiliations:** 1 Faculty of Food Technology, Josip Juraj Strossmayer University of Osijek, Franje Kuhača 18, 31000 Osijek, Croatia; tihomir.kovac@ptfos.hr (T.K.); ante.loncaric@ptfos.hr (A.L.); maja.izakovic@ptfos.hr (M.I.); 2 BC Institute for production and Field Crops, Dugoselska 7, 10370 Rugvica, Croatia; kjukic@bc-institut.hr

**Keywords:** high-voltage electric discharge (HVED) pre-treatment, wheat, germination, drought, salinity

## Abstract

The environmentally friendly, physical method of high voltage electrical discharge (HVED) was developed to improve the drought and salinity tolerance of two wheat genotypes. Unlike other plasma technologies, HVED treatment involves the discharge of electricity in water. In this study, the effect of HVED pretreatment on wheat germination and early vegetative growth under drought (0%, 15%, 20% and 30% PEG) and salinity (0, 90, 160 and 230 mM NaCl) stress conditions was investigated. HVED-exposed seeds showed altered seed surfaces and became more permeable to water uptake, resulting in higher germination percentages, germination index values, and shoot and root growth under the control and all drought and salinity concentrations. Moreover, the electrical conductivity of the water medium increased significantly, indicating HVED-induced reactions of ionization and dissociations of water molecules occurred. In addition, HVED pretreatment in the salt experiment improved the tolerance index values of the shoots and roots. The most pronounced genotypic variations occurred under the highest stress levels (30% PEG or 230 mM NaCl) and varied with the stress intensity and growth stage. The study results indicate that HVED pretreatment has the potential to improve drought and salt tolerance in wheat.

## 1. Introduction

Wheat is considered one of the most important cereals since it represents major staple food for 30% of the world’s population [1]. It is cultivated all over the world under various climatic and environmental conditions [2]. Current climate changes, which are mainly evident through global warming, are increasing the intensity of the abiotic stresses that significantly affect wheat production [3,4]. To date, many efforts have been made to mitigate the effects of drought and salinity on plant growth and development [5,6]. Numerous studies emphasize the negative connection between wheat growth and drought [7,8,9] or salinity [10,11,12].

To develop highly profitable genotypes with a wide tolerance range to nature’s impacts, scientists have been forced to find low-cost techniques that can successfully respond to extreme climate fluctuations. Since traditional methods for improving germination are not always sustainable, economically acceptable, and ecologically friendly physical methods, such as electric and magnetic field treatments, have the potential to improve plant tolerance for different kinds of environmental stressors. The mechanical treatments involve negligible or no damage on the seed surface, causing earlier germination despite an unpleasant environment. Furthermore, the use of these methods does not involve the presence of chemicals, making them safe for the environment and human health [13,14].

The high-voltage electrical discharge (HVED) method is a non-thermal technique that has application in food industry waste products but it can also be used as an extraction method in different industries, because it can alter the permeability of cell membranes and thus increase the overall membrane transport [15]. During the treatment, an electrical discharge occurs between electrodes immersed in an aqueous solution under the influence of a high voltage current. The electrical discharge leads to the formation of reactive oxygen species (ROS), which changes the ionic composition of liquid systems [16]. HVED application is based on the electrohydraulic discharge of water, which occurs along with several secondary phenomena, such as the formation of fluid turbulence, bubble cavitation, and high-pressure shock. The cavitated bubbles represent a major source of UV emission and ROS [17,18]. Furthermore, since HVED treatment generates ultraviolet light and shock waves, it is also suitable for disinfection [19,20].

Initially, drought and salinity have a similar osmotic effect on plants, decreasing cell water content and leading to loss of turgor [21]. The water imbalance leads to altered cellular homeostasis and the altered physiological and metabolic status of the plant, and eventually impaired plant growth [22,23]. The plant response to osmotic stress is a multicomponent strategy leading to reduced shoot growth, a stronger and longer root system, and earlier senescence of the older leaves [24]. Under drought, roots continue to grow and supply the upper parts with groundwater despite the water deficit, while in salt environments, they specialize in the accumulation of chloride to restrict its transport to the shoot [25]. Drought and salinity promote the excessive accumulation of ROS such as hydrogen peroxide (H_2_O_2_), hydroxyl radicals (HO·), and superoxide radicals (O_2_^−^). ROS excessive accumulation can act in several directions, including the damage of biomembranes and DNA, protein denaturation, enzyme activity inhibition, and even cell death [26,27]. Apart from causing osmotic imbalance, salinity implies an ionic effect, making it more destructive for plant survival. The extreme presence of intercellular NaCl hinders the nutrient availability and biochemical processes in the cell. Under elevated salinity, the water flux, besides important nutrient compounds, contains dissolved Na^+^ and Cl^−^ ions, which, as the transpiration stream and transport continues, move forward from root to shoots. Glycophyte plants, such as wheat (with a tolerance range of 50–100 mM NaCl), develop special strategies to cope with toxic Na^+^ and Cl^−^ levels [28]. Glycophyte plants mostly use compartmentation, meaning the Na^+^ intake across tonoplast Na^+^/H^+^ antiporters into vacuole to reduce toxic NaCl accumulation. Na^+^ exclusion is more common in halophyte plants, and involves the high activity of Na^+^/H^+^ plasma membrane antiporters and vacuolar sequestration [29]. However, the degree of effectiveness of a specific adaptive response to salinity varies among both salt-tolerance category groups and species [25].

The aim of this study was to explore the HVED impact on wheat germination and early vegetative growth (during the first 5 days) under control conditions, drought (0%, 15%, 20%, and 30% PEG), and salinity (0, 90, 160, and 230 mM NaCl) in two winter wheat genotypes. We also wanted to determine if there were differences between genotypes regarding HVED pre-treatment under salt stress and drought, and to determine whether the HVED had a similar impact on both stress conditions. The results provide, for the first time, the implementation of HVED treatment in abiotic stress studies.

## 2. Results

### 2.1. SEM Analysis of Seeds and Electric Conductivity Test

**Figure 1:**
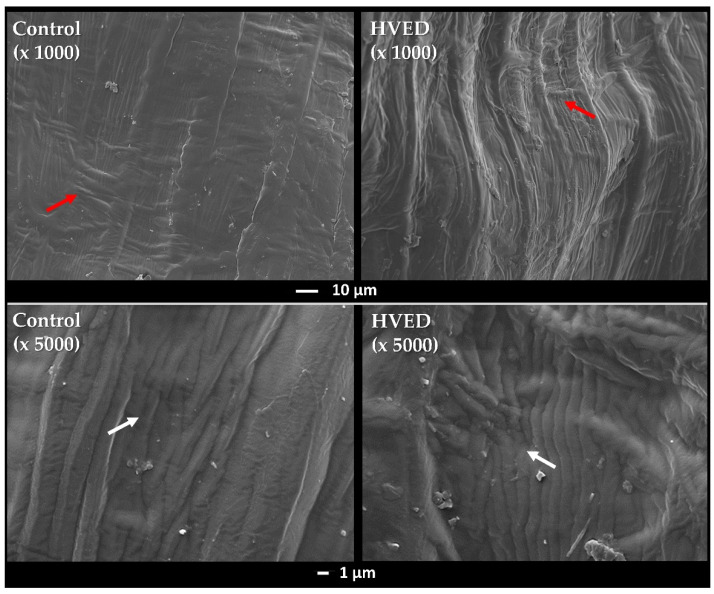
Selected scanning electron microscopy images of the seed morphology recorded at working distance of 10 mm with an acceleration voltage of 5 kV and magnifications of 1000× and 5000×. As a result of high voltage electrical discharge treatment (HVED), the number of mesh interconnected structures reduced (red arrows) and the wrinkles become more noticeable (white arrows).

The topology of the seeds before and after the HVED application is illustrated in Figure 1. It can be observed that the surface of the untreated seeds was covered with mesh interconnected structures. Contrarily, in HVED treated seeds, the mesh structures were less noticeable, and many wrinkles were noticed on the grain surface. The results of electric conductivity measurements revealed significant improvement in the water conductivity medium after the HVED application in both genotypes in regards to the control (Table 1).

### 2.2. Germination Parameters and Morphology under Drought before and after HVED Pretreatment

Analysis of variance was performed to determinate the interaction between drought treatments (T) and HVED pretreatment (*p*) for the germination percentage (G1–G4), germination index (GI), shoot length (S2, S3, and S5), root length (R) drought tolerance index for the shoot (SL_DT) and root (RL_DT) (Table S1). Genotype (G), drought treatment (T), HVED pretreatment (P) and interactions (G × P, G × T, T × P, and G × T × P) had a significant impact on all parameters. Significant interactions were not found among G × P for shoot length (S5).

**Figure 2:**
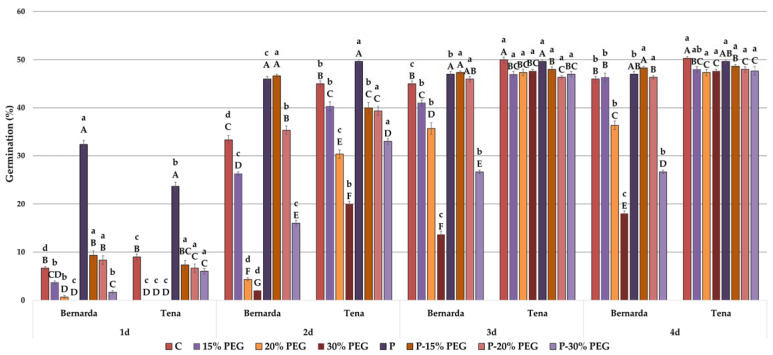
Germination percentage of two wheat genotypes during 4 days under control (C), drought treatments (15%, 20% and 30% PEG), high voltage electrical discharge (HVED) pre-treatment (P) and combined (P-15% PEG, P-20% PEG and P-30% PEG) treatments. Values are means of three repetitions (100 seeds/repetition) ± S.D. The different capital letters indicate significances among means of different treatments within the same genotype, while lowercase letters indicate significances among means and between genotypes under the same treatment at *p* < 0.05 using LSD post hoc-test.

The inhibitory drought effect on the germination (Figure 2) and GI (Table 2) was less pronounced in the HVED group than in the WP group in both genotypes at the early time points. However, after the 3rd day, no significance was found for the germination of Tena under drought treatments between the WP and HVED group, indicating the time-dependent response to drought. In Tena, HVED’s effect on germination was less visible under drought during this time, while at the same time, the Bernarda HVED group managed to retain a higher germination percentage than the WP group at 20% and 30% PEG, respectively. Comparing germination among genotypes in the WP group of plants, Bernarda started (G1) to germinate earlier than Tena under drought (15% and 20% PEG), but over time (G2–G4), the germination percentage in Tena significantly increased under control and drought levels. In the HVED group, a significant difference between genotypes was observed on the 2nd day of the experiment, where the germination of Bernarda was 16.75% higher than that of Tena under 15% PEG treatment, indicating that HVED could promote germination under mild (15% PEG) drought in Bernarda. The germination index of drought resistance (GIDR) is a common indicator of the drought tolerance of seeds, whereas its higher general values refer to better resistance. In the HVED group of Bernarda, the GIDR showed higher values as the drought intensity increased (Table 3). The increase of GIDR in the HVED group was 53.2% (20% PEG) to 66.2% higher (30% PEG) compared to the WP group, suggesting that HVED contributes to the drought resistance of Bernarda germination. However, GIDR values after HVED did not change under 15% PEG treatment, indicating that these commercial genotypes were able to tolerate the mild drought. The GIDR revealed genotype variations. Tena exhibited higher GIDR values than Bernarda at the highest drought level in both the WP (2.5 fold) and HVED (1.3 fold) groups. The results of the shoot and root growth under drought are presented in Table 4 and Table 5. HVED promoted shoot and root growth under the control and drought compared to the WP plants. Shoots were significantly higher in both genotypes in each sampling point. Based on the shoot and root lengths measured on the 5th day, shoot (SL_DT) and root (RL_DT) drought tolerance indices were calculated to reveal the differences between the WP and HVED groups under drought (Table 6). In Bernarda, the SL_DT value increased with the drought intensity. The HVED group also exhibited increased root growth. In Bernarda, the root lengths were 10% (control), 28% (15% and 30% PEG), and 45% higher (20% PEG) than in the WP group, while in Tena, HVED promoted growth under mild drought intensity (15% PEG). RL_DT values confirmed the significant improvement in drought tolerance of Bernarda in the HVED group under all drought treatments. Correlation analyses revealed a strong positive connection (*p* < 0.001) among drought tolerance indices (SL_DTI and RL_DTI) and G1–G4, S3, S5, or R, respectively (Table 7). Comparing the shoot growth values from the same plant group (WP or HVED), the same pattern was observed during the experiment’s entire duration. In both groups (WP and HVED), shoots of Tena were higher on the 2nd day, but that trend weakened overtime, and Bernarda finally managed to overgrow Tena (5th day). For instance, in the HVED group, the shoot growth of Tena on the 2nd day increased from 20% (control) to 89% (30% PEG treatment) in comparison to Bernarda. On the other hand, on the 5th day, Bernarda shoot growth increased by 13% (0% and 30% PEG concentrations) and 28% (15% PEG concentrations). All this suggests that HVED did not change the genotype growing rate properties. The main difference in root growth between genotypes was observed in the HVED group. The roots in Bernarda were approximately two times longer than those in Tena under the control and all drought treatments. In Bernarda, the RL_DT index ranged from 11.1% (15% PEG concentration) to 45% (20% PEG concentration) in comparison to Tena.

### 2.3. PCA Analysis for Drought Stress

**Figure 3:**
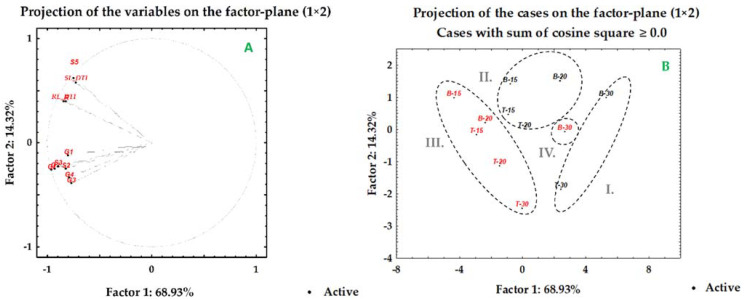
Principal component analysis of data sets of germination percentages (G), germination index (GI), shoot (S) and root (R) lengths, drought tolerance index for shoot (SL_DTI) and root (RL_DTI) lengths under drought (15%, 20% and 30% PEG) and combined (P-15% PEG, P-20% PEG and P-30% PEG) treatments. Genotypes Bernarda (B) and Tena (T). Factor loadings (**A**) and scores (**B**) of first two factors. Numbers beside each variable represent the day of treatment. Red marks present high voltage electrical discharge (HVED) pretreatment.

Figure 3A presents the percentages of the total variance and principal components (PC) and their interactions with germination (G1–G4, GI) and morphological (S2, S3, S5, R, SL_DT, SR_DT) parameters in the drought experiment. The PCA presents the total variation described by two components (PC1 and PC2), contributing 83.3% of the data variation under drought and combined HVED-drought treatments (Figure 3A). The total variation of the first principal component was 68.9%, and the second principal component was 14.3% of the data variation (Table S2). PC1 was characterized by strong negative loadings for all variables. Positive loadings were obtained in PC2 for shoot parameters (S5 and SL_DTI). The visualization of plots revealed four clusters under drought (Figure 3B). Cluster I belonged to the WP group of plants exposed to the highest drought level (30% PEG), which showed decreased germination, shoot and root growth inhibition and lower drought tolerance indices (SL_DTI, RL_DTI). Group II discriminated low (15% PEG) and medium (20% PEG) drought intensity for both Bernarda and Tena, indicating a similar pattern response in dealing with drought conditions. The HVED pre-treated plants were segregated into cluster III due to their enhanced germination, growth promotion, and higher drought tolerance index values. The HVED plants of Bernarda under 30% PEG (B − 30) belonged to cluster IV due to their increased shoot and root lengths (5th day) but lower germination percentages than Tena (T − 30).

### 2.4. Germination Parameters and Morphology under Salt Stress before and after HVED Treatment

To indicate connections among the salt treatments (T) and HVED pretreatment (P) for germination percentages (G1–G4), the germination index (GI), shoot length (S2, S3, and S5), root length (R), the salt tolerance germination index (STI_G1–G4), the salt tolerance shoot index (STI_S2, STI_S3, and STI_S5) and the salt tolerance root index (STI_R5), analysis of variance was conducted (Tables S3 and S4). Genotype (G), salt treatment (T), and HVED pretreatment (P) showed a significant impact on all parameters. This implied similar responses to salt stress. Considering G × P, G × T, T × P and G × T × P interactions, significances were observed for all variables except for G2, S5, and STI_G2 (G × P) and STI_R (G × T × P).

**Figure 4:**
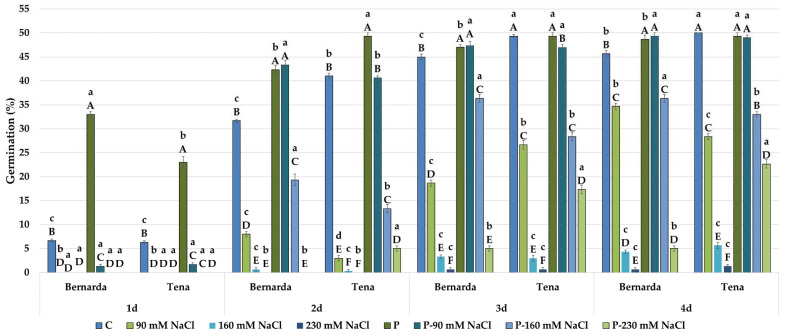
Germination percentages of two wheat genotypes during 4 days under control (C), salt treatments (90, 160 and 230 mM NaCl), high voltage electrical discharge (HVED) pretreatment (P) and combined (P-90 mM NaCl, P-160 mM NaCl and P-230 mM NaCl) treatments. Values are means of three repetitions (100 seeds/repetition) ± S.D. The different capital letters indicate significances among means of different treatments within the same genotype, while lowercase letters indicate significances among means between genotypes under the same treatment at *p* < 0.05 using LSD post hoc-test.

**Figure 5:**
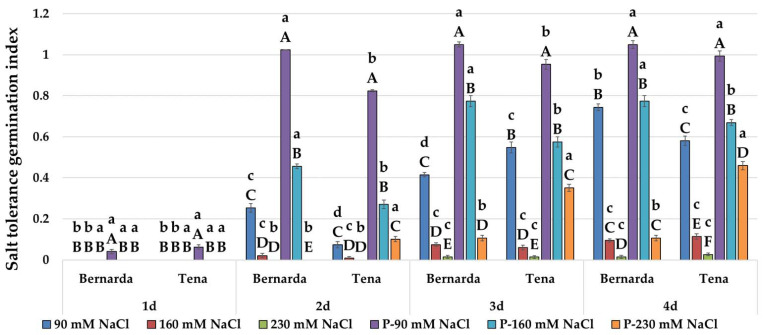
Salt tolerance germination index (STI_G) of two wheat genotypes during 4 days under salt treatments (90, 160 and 230 mM NaCl), high voltage electrical discharge (HVED) pretreatment (P) and combined (P-90 mM NaCl, P-160 mM NaCl and P-230 mM NaCl) treatments. Values are means of three repetitions (100 seeds/repetition) ± S.D. The different capital letters indicate significances among means of different treatments within the same genotype, while lowercase letters indicate significances among means between genotypes under the same treatment at *p* < 0.05 using LSD post hoc-test.

Analysing the significances between HVED and the corresponding WP group, it was evident that the HVED group of both genotypes showed significantly higher germination percentages (Figure 4) and germination index (Table 2) and salt tolerance germination index (STI_G) values under salt treatments (Figure 5). For, instance, STI_G in the HVED group increased from 0.62 to 16.24 times (in Tena) and 0.41 to 6.35 times (in Bernarda) as the salt concentration increased. HVED also had a beneficial effect on shoot and root growth promotion. For shoots and roots, the salt tolerance shoot index (STI_S) and root index (STI_R) values were higher in the HVED group exposed to 90, 160, and 230 mM NaCl during the experiment’s duration (Table 8). The same trend was noticed in the control groups for all morphological and germination parameters. The results of the correlation analyses showed that the salt tolerance index of germination (STI_G) strongly depended on the germination day (Table 9). The *r*-values between STI_G and corresponding germination days were 0.75 (G1), 0.55 (G2), 0.50 (G3), and 0.57 (G4) at *p* < 0.01 and 0.0001 levels. The variation between genotypes in response to different salt concentrations was most notable in the HVED group. The HVED effect became visible on the 2nd day of treatment, because thereupon Bernarda germination increased under lower salt concentrations (90 and 160 mM NaCl) (Figure 4). Contrarily, the HVED effect was more efficient in Tena at the highest salt concentration (230 mM NaCl). The Tena HVED group exhibited higher germination (3.53 times), GI (4.4 times), and STI_G (1.6 times) values than Bernarda on the 4th day of the experiment. At the higher salt concentrations (160 and 230 mM NaCl), for the WP group, both of the genotypes showed the same pattern for root and shoot growth (Table 4 and Table 5), as well as for the STI_S or STI_R parameters (Table 8) with regards to the similarity in salt tolerance (from the 3rd and 5th day). Contrarily, within the HVED group, Bernarda showed better shoot (50% at 90 mM NaCl and 22% higher at 160 mM NaCl) and root growth (27% at 90 mM NaCl or 6.8% higher at 160 mM NaCl) than Tena. However, HVED improved the Tena shoot and root growth, STI_S, and STI_R under severe salt stress (230 mM NaCl), indicating a shift in its tolerance response to the extreme salt environment.

### 2.5. PCA Analysis for Salt Stress

**Figure 6:**
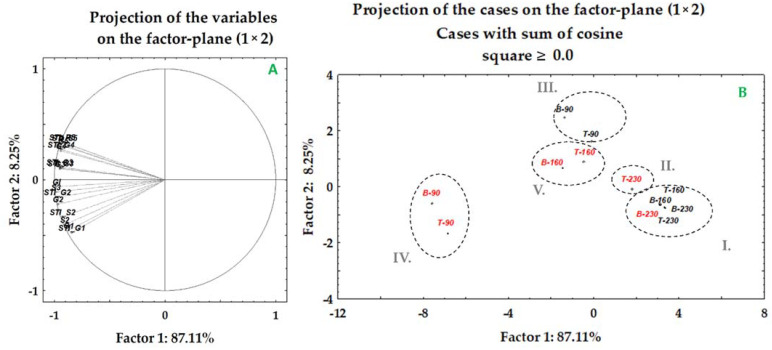
Principal component analysis of data sets of germination percentages (G), the germination index (GI), shoot (S) and root (R) lengths, and salt tolerance indices for germination (STI_G), shoot (STI_DT) and root (STI_R) lengths under salt treatments (90, 16 and 230 mM) and combined (P-90 mM, P-160 mM and P-230 mM NaCl) treatments. Genotypes Bernarda (B) and Tena (T). Factor loadings (**A**) and scores (**B**) of first two factors. Numbers beside each variable represent the day of treatment. Red marks represent high voltage electrical discharge (HVED) pretreatment.

Figure 6A summarizes the PCA grouping among the WP and HVED groups under different salt stress treatments. A variable projection is given for the first two components, contributing 95% of the total variation under salt treatments. The most important component (PC1) showed an 87.1% cumulative contribution to the total variation (Table S5). A negative strong association was found for most of the germination parameters (G2–G4, GI, S2, S3, S5, R) and the salt tolerance indices of the shoots (STI_S2-STI_S4) and roots (STI_R5). The second component was determined by negative loadings of the germination percentages (G1) and salt tolerance index of germination (STI_G1) on the 1st day. The corresponding score plot shows five groups (Figure 6B). The first group included WP plants of Tena and Bernarda due to their strong inhibition of germination (G2–G4), the germination index, morphometry parameters (S2, S3, S5, R), and salt tolerance indices of the shoots (STI_S2, STI_S3, STI_S5), roots (STI_R5) and germination (STI_G2–STI_G4) under medium (160 mM) and extreme (230 mM) NaCl concentrations. Unexpectedly, the HVED-pretreated plants of Bernarda (B − 230) were grouped into the same cluster (cluster I) due to their slow shoot and root growth and poor salt tolerance index values with regards to shoot and root growth. Cluster II discriminated Tena (T − 230) from Bernarda (B − 230) under the same treatment combination. Unlike Bernarda, Tena (T − 230) displayed a great increase in the salt tolerance index values for germination (STI_G2–STI_G4), shoots (STI_S3, STI_5), roots (STI_R5), shoot lengths (S3, S5), and root lengths (R), which implies that HVED pretreatment application improved the salt tolerance of Tena under high salt concentrations. The lowest salt concentration (90 mM NaCl) grouped both genotypes into the same cluster III due to the similar salt tolerance of WP plants of Bernarda and Tena. The HVED-pretreated plants grown under 90 mM NaCl were grouped into the cluster IV based on their promoted growth and germination as compared to cluster III. Cluster IV recorded increased germination and germination index values, promoted shoot and root growth, and had higher salt tolerance index values for germination, shoots, and roots. HVED-treated Tena and Bernarda plants under 160 mM NaCl were grouped into cluster V due to their germination salt tolerance index values (STI_G2–STI_G4), germination (G2–G4), shoot (S2, S3, and S5) and root (R) growth parameters, and salt tolerance index values for shoots (STI_S2, STI_S3, and STI_S5) and roots (STI_R5), which were all higher than those recorded for cluster I.

### 2.6. Comparative Statistical Analyses

To reveal the connections among the effects of the HVED pretreatment and treatments on the germination and morphometric parameters during all five days, comparative statistical analyses were performed for both drought and salt stress (Table 7 and Table 9). In both stress types, HVED showed a positive correlation for all germination parameters and for shoot length. On the contrary, drought and salt treatments showed a strong negative correlation with germination, shoot and root lengths, drought tolerance indices (SL_DTI, RL_DTI), and salt tolerance indices (STI_G, STI_S, and STI_R). For both stresses, the same correlation test showed a strong association between the tolerance indices of the two plant organs on the 5th day of growth. In the drought experiment, the correlation value (*r*) between the drought tolerance indices (SL_DTI and RL_DTI) was 0.75, and under salt stress, the salt tolerance shoot (STI_S5) and root indices (STI_R5) exhibited an even higher connection (*r* = 0.98).

## 3. Discussion

Physical methods for improving seed germination, such as cold atmospheric pressure plasma technology [30,31], low voltage electrical discharge [32], pulsed magnetic field [33], and UV-A rays [34] have been widely applied in agricultural and biological studies. Plasma technology uses different working gas sources (Ar, Ne, N_2_, O_2_, air, and the combination of gasses) to generate plasma. Electrical discharge treatment has been shown to cause the dissociation of water to OH^.^ and H^.^ as well as modifications in the ionization and vibrational/rotational excitation of water molecules [35]. In this study, the HVED technology was used, and reactions of ionization and the dissociation of water molecules occurred, as well as seed surface modifications. Although the HVED technology differs in configuration, frequency, power, and voltage across most plasma devices, its effect on grain is very similar. Our results describe for the first time the effect of HVED on wheat germination and growth under drought and salt stress conditions.

### 3.1. The Impact of HVED on Germination under Drought and Salt Stress

A wide range of evidence describes the role of different plasma treatments in germination and growth promotion of various plant species under optimal conditions [13,36,37,38]. However, limited information is available in the literature describing the drought–plasma or salinity–plasma interaction. In our study, HVED promoted germination percentages and germination index values under the control and drought treatments in both genotypes, especially at the beginning of the experiment (Figure 2 and Table 2). The correlation analyses showed a strong positive correlation between HVED and G1 (*r* = 0.81, *p* < 0.001) and G2 (*r* = 0.52, *p* < 0.01) (Table 7). The benefits of HVED application on germination percentages (Figure 4) and the germination index (Table 2), and salt tolerance germination index values (STI_G) (Figure 5) were also noticed under the control and salt treatments. Germination parameters were positively correlated with HVED, whereas the WP group was negatively correlated with salt treatments (Table 9). The promotion of germination by HVED might be associated with the mechanical damage of seed shells, creating a humid environment. SEM micrographs of the seed coat also confirmed the surface abrasion (Figure 1). This supports evidence that the etching effect of plasma evokes wax removal from seed surfaces [39]. However, Fourier-transform infrared spectroscopy (FTIR) analyses revealed that plasma-treated seed surfaces showed a decrease in the lipid component and an increase in nitrogen, oxygen, and oxygen-containing species attached to the hydrophilic part of the test [40]. Bousetta et al. [17] found in their study that HVED application successfully disrupted plant tissues. In the same study, wheat seeds showed better wettability after air plasma application. A large number of studies show that short-term physical treatments do cause ruptures on the seed surface and make water-impermeable coats permeable for water absorption [41,42,43]. The outer layers of the seed coat participate in dormancy expression. When the seed structure is altered, the diffusion of oxygen into the seed is faster, and germination is better [44]. This effect occurs in a gas environment. The HVED technology uses a liquid medium, which means that the better germination of pretreated seeds could be ascribed to the faster diffusion of dissolved oxygen and earlier embryo development. Electronic transitions in the plasma discharge generate UV light, ROS, and reactive nitrogen species (RNS) production, which disrupts the seed dormancy [45,46]. UV emissions, released during plasma technology treatments, trigger an earlier germination, but also can have a mutagen effect, causing DNA (de)methylation and alterations in gene expression [39]. Surprisingly, this is a rare scenario in plant cells. One of the possible explanations is their rigid cell walls and seed coats, which create a mechanical barrier [39]. The second reason might be connected with the activation of DNA-repairing mechanisms induced by nonthermal plasma (NTP) [47]. In [47], NTP did not cause DNA damage in pea seeds. Finally, the seed exposure time to HVED was short (30 s), and despite the high voltage dose (30 kV), we can assume that the embryo cells’ genome remained intact. Our results showed increased water conductivity (Table 1) after the HVED treatment, suggesting the changes occurred in the chemical properties of medium and ROS and RNS formation, resulting in higher germination percentages. The electroconductivity of seeds was also improved after the application of air plasma in several studies [45,48]. The positive effect of different types of plasma priming on germination parameters under optimal conditions has been previously noticed in wheat [30,35,40], rye [49], and legumes [42]. Several studies have reported increased germination under drought in wheat [13], alfalfa [50], and rapeseed (*Brassica napus* L.) [51]. Sheteiwy et al. [52] reported beneficial cold plasma effects on germination, seedling vigor index values, shoot, and root lengths, and the activity of antioxidative enzymes (CAT, APX, SOD, and POD) in rice under 150 mM NaCl.

In this study, the various genotype responses in the HVED group were recorded for both stresses. Immediately after HVED application (1st day), Tena showed higher germination percentages than Bernarda (73%) (Figure 2) and higher GIDR values (approx. 9.9%) at 30% PEG (Table 3). Similar behaviour was noticed in the salt experiment. Tena started to germinate earlier (2nd day, at 230 mM NaCl) (Figure 4) and obtained higher GI (Table 2) and STI_G2 (Figure 5) values than those of Bernarda, which may be connected with their different seed topography. The best effects of plasma on germination in different crops depend on seed size, seed coat hardness, and the presence of keratin, suberin, or lignin [30,41,53]. In our case, the differences in germination imply that HVED application outcomes may be related to the different porosities of genotype seed coats. Thus, we can assume that Tena has a thinner seed coat than Bernarda, which disrupts faster after HVED application, creating an optimal water gradient for water uptake and earlier germination under intense drought or salinity. However, further investigation in this direction is needed.

### 3.2. The Impact of HVED on Growth under Drought and Salt Stress

In general, plants treated with HVED were less vulnerable to drought or salinity than those that were not treated (WP group). The HVED-treated plants displayed longer shoots and roots (Table 3 and Table 4), salt tolerance index values (Figure 5 and Table 8), and drought tolerance index values (for Bernarda only) (Table 6). Knowing that drought and salt stress initially causes cell osmotic imbalance, it can be assumed that HVED increases a plant’s water uptake. Similar findings have reported that plasma technology triggers the water balance regulatory pathways, causing growth promotion, although the exact mechanism has not yet been explored. Guo et al. [13] reported a positive correlation among the expression of the drought-responsible genes *SnRK2* and pyrroline−5-carboxylate synthase (*P5CS*) involved in proline biosynthesis with abscisic acid (ABA) signalling routes in wheat after NTP treatment. Furthermore, some studies amplified the positive plasma impact on cellular antioxidant systems under abiotic stress, although the activity of certain enzymes varied in terms of the intensity, species, developmental age and plasma treatment parameters [51,54,55]. Cold plasma treatment (CPT) has been found to accelerate the growth of alfalfa [50] and oilseed rape [51] under drought conditions (15% PEG). Moreover, in a salt stress study, CPT showed the potential to re-establish cell turgidity in rice [52]. Further, in another study, NTP technology, combined with salinity, induced the expression of heat shock factor A4A (HSFA4A) in roots and shoots, enhanced the biomass, alleviated chlorophyll degradation, and increased phenylalanine ammonia-lyase (PAL) in wheat [54]. In our study, the HVED-treated group of plants in both genotypes managed to grow despite extreme salt concentrations (160 and 230 mM NaCl) as compared to the WP group, which leads us to the conclusion that HVED raises the salt tolerance range (Table 8). This effect was less visible in the drought experiment, suggesting that the success of HVED application depends on the stress type, regardless of the plant species. Several studies have reported significant improvements in root and shoot growth after exposure to various physical treatments. Radish shoots were over 60% higher after an oxygen RF plasma treatment [56], and the fresh (10%) and dry (14%) weights of maize were enhanced after treatment with low-temperature plasma [57].

Shoot and root growth kinetic variations between the two genotypes under the drought and salt experiments were observed. At the beginning of the experiment (2nd day), Tena had longer shoots than Bernarda’s under all drought treatments, but as the experiment progressed (5th day), Bernarda’s shoots overtook Tena’s (Table 4). Under the highest drought level, the cluster analyses separated Tena from Bernarda (Figure 3B) due to the different HVED effects on germination (Figure 2), shoot and root lengths (Table 4 and Table 5), and RL_DTI (Table 6), suggesting that Bernarda had better drought tolerance. However, in the drought experiment, the HVED outcomes varied with time, the growth stage, and the drought intensity. Similar responses were noticed in the salt experiment, where Bernarda shoots and roots were longer after the HVED pretreatment (at 90 and 160 mM NaCl) than Tena’s (Table 4 and Table 5). On the contrary, the combination of HVED and the highest salt concentration (230 mM NaCl) stimulated germination (Figure 4) and the shoot (Table 4) and root (Table 5) growth of Tena, indicating that HVED could be an important strategy in elevating the tolerance of salt-sensitive genotypes. The PCA analyses separated the two genotypes according to their responses at the highest salt concentration (Figure 6B). These unexpected results demand further investigation.

## 4. Materials and Methods

### 4.1. HVED System and Seed Pretreatment

**Figure 7:**
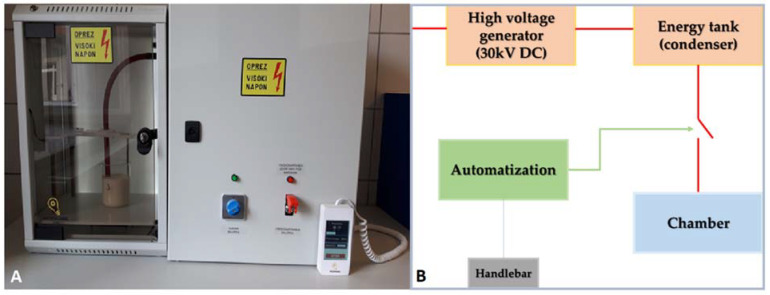
The high-voltage electrical discharge (HVED) device used in the study (**A**) and a basic HVED schematic diagram (**B**).

HVED is a non-thermal method that is performed on a device consisting of a high-voltage pulse generator chamber connected to a chamber with a constant discharge voltage of 30 kV and a changeable frequency range of 10–100 Hz (Figure 7). The chamber consists of a cylindrical needle (diameter 2.5 mm) and a plate electrode with grounding (diameter 4.5 cm). Demineralized water is used as a dielectric medium. The specifications and work principles of the HVED device have been described by Barišić et al. [16].

A total of 30 g of non-sterilized seeds was immersed into 800 mL of demineralized H_2_O, placed in a chamber, and subjected to HVED pretreatment for 30 Hz for 30 s with constant stirring. During all treatments, electrodes were spaced 2 cm apart. Seeds without HVED pretreatment represented the control (WP). The seeds of the control group (WP) were soaked in demineralized water for as long as the HVED group was exposed to HVED treatment (30 s).

### 4.2. Plant Material and Drought or Salinity Stress Application

In this study, two commercial winter wheat genotypes (BC Bernarda and BC Tena, produced at the BC Institute for Breeding and Production of Field Crops, Croatia) were used. Bernarda and Tena are commercial genotypes whose tolerance to drought or salinity has not been tested before. Drought and salinity stress tolerances of the genotypes were tested in two independent experiments under the same experimental conditions.

WP and HVED seeds were planted in transparent plastic pots (20 cm L × 15 cm W × 3 cm H) on two layers of filter paper containing 15%, 20% and 30% PEG − 6000 (polyethylene glycol) (*w*/*v*) or 0, 90, 160 and 230 mM NaCl, respectively. Demineralized water without PEG or NaCl was used as a control. To prevent evaporation, plastic containers were covered and watered every two days with 10 mL of corresponding PEG or NaCl concentrations. One hundred seeds in three replications of each genotype for drought and salt stress were used. Seeds were kept in darkness at room temperature (approx. 22 °C) in a climate chamber, and after 48 h were exposed to 12 h light/12 h dark photoperiods and an artificial fluorescent lamp (80 mol m − 2 s − 1) at 22 ± 2 °C for five days.

### 4.3. Plant Material and Drought or Salinity Stress Application

Seeds were considered germinated when the protrusion of radicles and coleoptiles occurred. Germinated plants were counted daily. The germination percentage was calculated according to Thabet et al. [58] (Equation (1)):

(1) G%=n/N × 100

n = number of germinated seeds;

N = number of total seeds per pot.

The germination index (GI) was determined by the following expression [13] (Equation (2)):

(2) GI=1 × GR1+0.75 × GR2+0.50 × GR3+0.25 × GR4

GR1, GR2, GR3 and GR4 = germination rates from 1st to 4th day after sowing.

The germination rate for each day was determined using the equation [59] (Equation (3)):

(3) GR=X/Y

X = germinated seeds per day;

Y = day that germination appeared.

To verify the effect of HVED pretreatment under the control and drought conditions on the 5th day of the experiment, the germination index of drought resistance (GIDR) was calculated by the following equation [13]:

(4) GIDR=GID/GIC 

GIC = germination index under the control;

GID = germination index under drought.

In the salt experiment, the salt tolerance index (STI) was expressed to evaluate the efficiency of HVED pretreatment on germination over four days and the shoot and root responses to NaCl according to [60]

(5) STI=(NaCl exposed plants)/(control plants)

### 4.4. Measurement of Morphometry Parameters

To monitor the dynamics of plant growth, shoot lengths were measured on the 2nd, 3rd, and 5th days, whilst root lengths were tracked on the 5th day of the experiment using a scaler ruler in both stress experiments. Shoot (SL) and root lengths (RL) were measured on ten representative plants for each pot and treatment. The drought tolerance index values for shoot (SL_DTI) and root lengths (RL_DTI) were calculated on the 5-day-old seedlings according to [58]

(6) S=(SL_drought)/(SL_control)×100

As for the germination, the salt tolerance index was used for the shoot (STI_S) and root lengths (STI_R) on the 5th day of growth.

### 4.5. Scanning Electron Microscope (SEM) Analysis

The surface morphology of WP and HVED-treated wheat seeds was determined by scanning electron microscopy (JEOL JSM−7800F model) under 5 kV acceleration voltage at a working distance of 10 mm. Prior to SEM analyses, seeds were coated with 10 nm Au/Pd (PECS II).

### 4.6. Electric Conductivity Determination

To determine the relative leakage of ions from the seeds, electric conductivity (EC) was carried out with a modified method of Pandey [61]. A total of 50 seeds of the WP and HVED groups were weighed, immersed in 10 mL of demineralized water, and incubated at room temperature. After 6 h, the conductivity (C) was measured (Mettler Toledo S30 SevenEasy). Three replications of each sample were taken, and demineralized water represented a blank. Electric conductivity was calculated by the following equation [48]:

(7) EC=((Csample−Cblank))/(seeds weight)

### 4.7. Data Analysis

Statistical analyses were performed using Statistica 13.5 (TIBCO Software Inc., Palo Alto, CA, USA). All separate treatments (control, HVED, PEG, and NaCl) and treatment combinations (HVED + PEG or HVED + NaCl) were performed in three pots per concentration. Results for both drought and salt stress were expressed as the mean value ± standard deviation. Factorial analysis of variance (ANOVA) was conducted to confirm the variability of the results and to explore the interactions between genotype (G), treatment (T), pretreatment (P), and combined G × T, G × P, T × P, and G × T × P. Differences among the variables (G, T, P) were compared using Fisher’s least significant difference (LSD) test. Spearman’s correlation coefficients were determined among all variables for drought and salt stress separately. Principal Component Analysis (PCA) was applied to visualize the separation of different treatment combinations. Factor loadings were performed to assess the proportion of total variance with different principal components (PC). The loadings showed the correlations between different PC and variables, where high values (>0.71) were referred to as strong [62].

## 5. Conclusions

In conclusion, HVED pre-treatment improved the germination percentages, germination index values, and shoot and root growth under control, drought, and salt conditions in both genotypes. Along with this, a better HVED stimulation effect was achieved in the salt experiment, under which the germination salt tolerance index values and shoots and roots salt tolerance index values were increased. Application of HVED revealed intergenotypical variance among wheat cultivars, especially at the highest stress level. The opposite effect was found for drought and salt stress. Under 30% PEG or 230 mM NaCl treatments, HVED led to higher germination percentages of Tena than those of Bernarda. On the contrary, Bernada root and shoots exhibited growth promotion at 30% PEG treatment, but growth inhibition under 230 mM NaCl. This means that the percentage of success of HVED treatment greatly depends on the stress type and intensity, the developmental stage, and the genotype properties. The study highlights the possible implementation of this technology for tolerance enhancement under salinity and drought stress. Its future prospects will be further elucidated after the molecular and metabolic response mechanisms become better understood.

## Figures and Tables

**Table 1:** The impact of high voltage electrical discharge (HVED) on the electrical conductivity of the water medium of wheat seeds. Values are means of three repetitions (50 seeds/repetition) ± S.D. The different letters indicate significances among means of different treatments and between genotypes under same treatment at *p* < 0.05 using LSD post hoc-test.

Pretreatment	Electrical Conductivity (µS/cm/g)	
	Bernarda	Tena
Control	66.6 ± 2.6 a	62.4 ± 2.9 a
HVED	91.8 ± 1.1 b	97.2 ± 2.1 b

**Table 2:** Germination index of two wheat genotypes during 4 days under control (C), drought (15%, 20% and 30% PEG), salt treatments (90, 160 and 230 mM NaCl), high voltage electrical discharge (HVED) pre-treatment (P) and combined (P-15% PEG, P-20% PEG, P-30% PEG, P-90 mM NaCl, P-160 mM NaCl and P-230 mM NaCl) treatments. Values are means of three repetitions (100 seeds/repetition) ± S.D. The different capital letters indicate significances among means of different treatments within the same genotype, while low case letters indicate significances among means between genotypes under the same treatment at *p* < 0.05 using LSD post hoc-test.

*Drought*			*Salt Treatment*		
	**Bernarda**	Tena		Bernarda	Tena
**C**	29.54 ± 0.5 ^dD^	37.35 ± 0.3 ^cB^	**C**	28.86 ± 0.3 ^dB^	32.72 ± 0.8 ^dB^
**15% PEG**	23.27 ± 0.6 ^dE^	25.96 ± 0.4 ^cE^	**90 mM NaCl**	8.24 ± 0.4 ^cD^	7.43 ± 0.5 ^cE^
**20% PEG**	10.51 ± 0.0 ^cG^	22.22 ± 0.5 ^bF^	**160 mM NaCl**	1.08 ± 0.2 ^cE^	0.98 ± 0.2 ^cF^
**30% PEG**	4.15 ± 0.1 ^dH^	18.42 ± 0.3 ^bG^	**230 mM NaCl**	0.15 ± 0.1 ^cE^	0.19 ± 0.1 ^cF^
**P**	58.98 ± 0.5 ^aA^	53.67 ± 0.9 ^bA^	**P**	59.65 ± 0.6 ^aA^	53.14 ± 1.2 ^bA^
**P-15% PEG**	37.74 ± 0.9 ^aB^	33.36 ± 0.6 ^bC^	**P-90 mM NaCl**	28.83 ± 0.7 ^aB^	26.15 ± 0.0 ^bC^
**P-20% PEG**	32.15 ± 0.8 ^aC^	32.14 ± 1.3 ^aC^	**P-160 mM NaCl**	15.58 ± 0.3 ^aC^	13.45 ± 0.8 ^dB^
**P-30% PEG**	13.78 ± 0.1 ^cF^	29.19 ± 0.8 ^aD^	**P-230 mM NaCl**	1.15 ± 0.1 ^bE^	6.18 ± 0.0 ^aE^

**Table 3:** Germination index of drought resistance (GIDR) in WP group of plants (without pre-treatment) and high voltage electrical discharge (HVED) pre-treated group under control and different PEG levels.

Treatment	WP Group		HVED	
PEG	Bernarda	Tena	Bernarda	Tena
**15%**	0.79	0.69	0.64	0.62
**20%**	0.36	0.59	0.55	0.60
**30%**	0.14	0.49	0.23	0.54

**Table 4:** Shoot length (mm) of two wheat genotypes at 2, 3 and 5 days of growth under control (C), drought treatments (15%, 20% and 30% PEG), salt treatments (90, 160 and 230 mM NaCl), high voltage electrical discharge (HVED) pretreatment (P) and combined (P-15% PEG, P-20% PEG, P-30% PEG, P-90 mM NaCl, P-160 mM NaCl and P-230 mM NaCl) treatments. Values are means of three repetitions (10 plants/repetition) ± S.D. The different capital letters indicate significances among means of different treatments within the same genotype, while lowercase letters indicate significances among means between genotypes under the same treatment at *p* < 0.05 using LSD post-hoc test.

*Drought*							*Salt Treatment*						
	2d		3d		5d			2d		3d		5d	
	Bernarda	Tena	Bernarda	Tena	Bernarda	Tena		Bernarda	Tena	Bernarda	Tena	Bernarda	Tena
**C**	5.2 ± 0.2 ^dB^	6.4 ± 0.1 ^bB^	13 ± 0.2 ^dD^	17.8 ± 0.2 ^cD^	84 ± 0.5 ^bB^	75.7 ± 0.3 ^dB^	**C**	5.7 ± 0.2 ^bB^	6.0 ± 0.2 ^bB^	13.2 ± 0.2 ^dC^	17.8 ± 0.2 ^cB^	82.9 ± 0.3 ^bB^	75.7 ± 0.3 ^dB^
**15% PEG**	3.5 ± 0.3 ^bCD^	2.7 ± 0.1 ^cE^	8.5 ± 0.5 ^cF^	12.8 ± 0.5 ^bF^	70.2 ± 0.6 ^bD^	58.0 ± 0.6 ^dD^	**90 mM NaCl**	0 ± 0.0 ^bE^	0 ± 0.0 ^bE^	5.1 ± 0.2 ^cD^	3.3 ± 0.1 ^dD^	41.7 ± 0.6 ^bD^	21.0 ± 0.4 ^dD^
**20% PEG**	0 ± 0.0 ^dE^	2.3 ±0.2 ^cE^	5.0 ± 0 ^cG^	12.4 ± 0.4 ^bF^	54.3 ± 0.7 ^bcF^	54.1 ± 0.3 ^abF^	**160 mM NaCl**	0 ± 0.0 ^bE^	0 ± 0.0 ^bE^	0 ± 0.0 ^cF^	0 ± 0.0 ^cF^	0 ± 0.0 ^cF^	0 ± 0.0 ^cG^
**30% PEG**	0 ± 0.0 ^cE^	0 ± 0.0 ^cF^	2.7 ± 0.1 ^dH^	8.2 ± 0.3 ^cG^	38.0 ± 0.6 ^aG^	21.3 ± 0.7 ^cH^	**230 mM NaCl**	0 ± 0.0 ^aE^	0 ± 0.0 ^aE^	0 ± 0.0 ^bF^	0 ± 0.0 ^bF^	0 ± 0.0 ^bF^	0 ± 0.0 ^cG^
**P**	5.8 ± 0.1 ^cA^	7.0 ± 0.1 ^aA^	30.8 ± 0.2 ^aA^	24.4 ± 0.2 ^bA^	91.7 ± 0.6 ^aA^	81.0 ± 0.2 ^cA^	**P**	6.2 ± 0.1 ^bA^	7.2 ± 0.1 ^aA^	31.0 ± 0.2 ^aA^	24.4 ± 0.2 ^bA^	91.1 ± 0.4 ^aA^	81.2 ± 0.2 ^cA^
**P-15% PEG**	5.8 ± 0.2 ^aA^	6.0 ± 0.1 ^aBC^	24.2 ± 0.1 ^aB^	23.2 ± 0.1 ^aB^	80.8 ± 0.7 ^aC^	63.0 ± 0.3 ^cC^	**P-90 mM NaCl**	3.0 ± 0.0 ^aC^	3.1 ± 0.1 ^aC^	14.7 ± 0.2 ^aB^	13.1 ± 0.2 ^bC^	52.3 ± 0.6 ^aC^	34.9 ± 0.3 ^cC^
**P-20% PEG**	3.8 ± 0.2 ^Bc^	5.3 ± 0.2 ^aD^	18.9 ± 0.3 ^aC^	19.4 ± 0.3 ^aC^	56.3 ± 0.8 ^aE^	56.1 ± 0.7 ^abE^	**P-160 mM NaCl**	1.0 ± 0.0 ^aD^	1.0 ± 0.0 ^aD^	3.6 ± 0.2 ^aE^	3.0 ± 0.0 ^bD^	18.6 ± 0.5 ^aE^	15.3 ± 0.3 ^bE^
**P-30% PEG**	3.0 ± 0.3 ^bD^	5.6 ± 0.2 ^aCD^	11.4 ± 0.3 ^cE^	15.1 ± 0.2 ^bE^	39.8 ± 0.6 ^aG^	35.1 ± 0.4 ^bG^	**P-230 mM NaCl**	0 ± 0.0 ^aE^	0 ± 0.0 ^aE^	0 ± 0.0 ^bF^	1.0 ± 0.0 ^aE^	0 ± 0.0 ^bF^	1.0 ± 0.0 ^aF^

**Table 5:** Root length (mm) of two wheat genotypes after 5 days of growth under control (C), drought treatments (15%, 20% and 30% PEG), salt treatments (90, 160 and 230 mM NaCl), high voltage electrical discharge (HVED) pretreatment (P) and combined (P-15% PEG, P-20% PEG, P-30% PEG, P-90 mM NaCl, P-160 mM NaCl and P-230 mM NaCl) treatments. Values are means of three repetitions (10 plants/repetition) ± S.D. The different capital letters indicate significances among means of different treatments within the same genotype, while lowercase letters indicate significances among means between genotypes under the same treatment at *p* < 0.05 using LSD post-hoc test.

*Drought*			*Salt Treatment*		
	Bernarda	Tena		Bernarda	Tena
**C**	100.64 ± 0.9 ^aB^	99.50 ± 0.5 ^bA^	**C**	100.73 ± 1.0 ^bB^	100.60 ± 1.5 ^bA^
**15% PEG**	77.60 ± 0.7 ^cD^	77.50 ± 0.9 ^cD^	**90 mM NaCl**	46.00 ± 0.6 ^cD^	35.40 ± 0.8 ^dC^
**20% PEG**	67.36 ± 1.1 ^bE^	66.10 ± 0.9 ^bD^	**160 mM NaCl**	0.00 ± 0.0 ^cF^	0.00 ± 0.0 ^cF^
**30% PEG**	46.75 ± 0.7 ^cG^	58.50 ± 0.9 ^aF^	**230 mM NaCl**	0.00 ± 0.0 ^bF^	0.00 ± 0.0 ^bF^
**P**	110.10 ± 0.7 ^aA^	99.50 ± 0.8 ^bA^	**P**	109.30 ± 1.2 ^aA^	100.90 ± 1.3 ^bA^
**P-15% PEG**	99.20 ± 0.8 ^aBC^	80.60 ± 0.6 ^bB^	**P-90 mM NaCl**	63.20 ± 0.8 ^aC^	49.70 ± 0.7 ^bB^
**P-20% PEG**	97.40 ± 0.5 ^aC^	60.90 ± 0.7 ^cE^	**P-160 mM NaCl**	30.00 ± 0.8 ^aE^	28.10 ± 0.9 ^bD^
**P-30% PEG**	59.90 ± 0.5 ^aF^	54.10 ± 0.7 ^bG^	**P-230 mM NaCl**	0.00 ± 0.0 ^bF^	3.30 ± 0.1 ^aE^

**Table 6:** Shoot (SL_DTI) and root (RL_DTI) length drought tolerance index of two wheat genotypes after 5 days of growth under drought treatments (15%, 20% and 30% PEG), combined (P-15% PEG, P-20% PEG and P-30% PEG) treatments, and high voltage electrical discharge (HVED) pre-treatment (P). Values are means of three repetitions (10 plants/repetition) ± S.D. The different capital letters indicate significances among means of different treatments within the same genotype, while lowercase letters indicate significances among means between genotypes under the same treatment at *p* < 0.05 using LSD post-hoc test.

*SL_DTI*			*RL_DTI*		
	Bernarda	Tena		Bernarda	Tena
**15% PEG**	83.57 ± 0.6 ^bB^	76.62 ± 0.8 ^cA^	**15% PEG**	77.56 + 0.9 ^cB^	77.91 ± 1.1 ^cB^
**20% PEG**	64.66 ± 0.9 ^bC^	71.48 ± 0.5 ^aB^	**20% PEG**	67.77 + 1.1 ^bC^	66.45 ± 0.9 ^bC^
**30% PEG**	45.27 ± 0.9 ^aE^	28.13 ± 0.9 ^bE^	**30% PEG**	45.96 + 0.3 ^cE^	58.80 ± 0.9 ^aD^
**P-15% PEG**	88.15 ± 0.9 ^aA^	77.78 ± 0.5 ^cA^	**P-15% PEG**	90.10 + 0.6 ^aA^	81.06 ± 0.9 ^bA^
**P-20% PEG**	61.44 ± 1.1 ^cD^	69.25 ± 0.8 ^aC^	**P-20% PEG**	88.51 + 0.8 ^aA^	61.22 ± 0.6 ^cD^
**P-30% PEG**	43.42 ± 0.8 ^aF^	43.33 ± 0.6 ^aD^	**P-30% PEG**	54.43 + 0.7 ^bD^	54.40 ± 0.8 ^bE^

**Table 7:** Spearman’s correlation coefficients (r-values) among germination (G), shoot (S) and root (R) length, shoot length drought tolerance index (SL_DT), root length drought tolerance index (RL_DT) under drought treatments and high voltage electrical discharge (HVED) pretreatment (*, **, ***, significant at the 0.05, 0.01 and 0.001 levels). Numbers next to capital letters represent the treatment day.

Treatment	HVED	G1	G2	G3	G4	S2	S3	S5	R	SL_DTI5	SL_DTI5
**Treatment**	1.000										
**HVED**	0.000 ns	1.000									
G1	−0.362 *	0.809 ***	1.000								
G2	−0.590 ***	0.522 **	0.656 ***	1.000							
G3	−0.502 **	0.233 ns	0.430 **	0.794 ***	1.000						
G4	−0.557 ***	0.188 ns	0.445 **	0.806 ***	0.987 ***	1.000					
S2	−0.430 **	0.731 ***	0.795 ***	0.805 ***	0.520 **	0.547 ***	1.000				
S3	−0.462 **	0.784 ***	0.807 ***	0.867 ***	0.613 ***	0.581 ***	0.807 ***	1.000			
S5	−0.906 ***	0.194 ns	0.506 **	0.537 **	0.289 ns	0.345 *	0.521 **	0.515 **	1.000		
R	−0.748 ***	0.296 ns	0.592 ***	0.591 ***	0.500 **	0.506 **	0.384 ns	0.601 ***	0.764 ***	1.000	
**SL_DTI5**	−0.953 ***	0.081 ns	0.400 *	0.565 ***	0.354 *	0.414 *	0.501 **	0.479 **	0.975 ***	0.695 ***	1.000
**SL_DTI5**	−0.831 ***	0.216 ns	0.553 ***	0.623 ***	0.582 ***	0.596 ***	0.396 *	0.591 ***	0.783 ***	0.976 ***	0.748 ***
		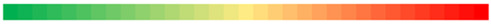									
		−1			0			1			

**Table 8:** Salt tolerance shoot index (STI_S) and salt tolerance root index (STI_R) of two wheat genotypes at 2, 3 and 5 days of growth under salt treatments (90, 160 and 230 mM NaCl), combined (P-90 mM NaCl, P-160 mM NaCl and P-230 mM NaCl) treatments, and high voltage electrical discharge (HVED) pretreatment (P). Values are means of three repetitions (100 seeds/repetition) ± S.D. The different capital letters indicate significances among means of different treatments within the same genotype, while lowercase letters indicate significances among means and between genotypes under the same treatment at *p* < 0.05 using LSD post hoc-test.

Treatment	*STI_S*						*STI_R*		
	2d		3d		5d			Bernarda	Tena
	Bernarda	Tena	Bernarda	Tena	Bernarda	Tena			
**90 mM NaCl**	0.00 + 0.0 ^bC^	0.00 + 0.0 ^bC^	0.39 + 0.0 ^cB^	0.19 + 0.0 ^dB^	0.50 + 0.0 ^bB^	0.28 + 0.0 ^dB^	**90 mM NaCl**	0.46 + 0.0 ^bB^	0.36 + 0.0 ^dB^
**160 mM NaCl**	0.00 + 0.0 ^bC^	0.00 + 0.0 ^bC^	0.00 + 0.0 ^cD^	0.00 + 0.0 ^cE^	0.00 + 0.0 ^cD^	0.00 + 0.0 ^cE^	**160 mM NaCl**	0.00 + 0.0 ^bD^	0.00 + 0.0 ^bE^
**230 mM NaCl**	0.00 + 0.0 ^aC^	0.00 + 0.0 ^aC^	0.00 + 0.0 ^bD^	0.00 + 0.0 ^bE^	0.00 + 0.0 ^bD^	0.00 + 0.0 ^bE^	**230 mM NaCl**	0.00 + 0.0 ^bD^	0.00 + 0.0 ^bE^
**P-90 mM NaCl**	0.42 + 0.0 ^aA^	0.43 + 0.0 ^aA^	0.63 + 0.0 ^aA^	0.54 + 0.0 ^bA^	0.57 + 0.0 ^aA^	0.43 + 0.0 ^cA^	**P-90 mM NaCl**	0.57 + 0.0 ^aA^	0.49 + 0.0 ^cA^
**P-160 mM NaCl**	0.14 + 0.0 ^aB^	0.14 + 0.0 ^aB^	0.16 + 0.0 ^aC^	0.12 + 0.0 ^bC^	0.20 + 0.0 ^aC^	0.19 + 0.0 ^bC^	**P-160 mM NaCl**	0.27 + 0.0 ^aC^	0.28 + 0.0 ^aC^
**P-230 mM NaCl**	0.00 + 0.0 ^aC^	0.00 + 0.0 ^aC^	0.00 + 0.0 ^bD^	0.04 + 0.0 ^aD^	0.00 + 0.0 ^bD^	0.01 + 0.0 ^aD^	**P-230 mM NaCl**	0.00 + 0.0 ^bD^	0.03 + 0.0 ^aD^

**Table 9:** Spearman’s correlation coefficients (r-values) among germination (G), shoot (S) and root (R) lengths, salt tolerance germination index (STI_G), and salt tolerance shoot (STI_S) and root (STI_R) indices under salt stress and high electrical discharge (HVED) pretreatment (*, **, ***, significant at 0.05, 0.01 and 0.001 level). Numbers next to capital letters represent the treatment day.

	Treatment	HVED	S2	S3	S5	R	G1	G2	G3	G4	GI	STI_S5	STI_R5	STI_G1	STI_G2	STI_G3	STI_G4
Treatment	1																
HVED	0.000	1															
S2	−0.550 ***	0.591 ***	1														
S3	−0.728 ***	0.463 **	0.931 ***	1													
S5	−0.853 ***	0.285	0.713 ***	0.882 ***	1												
R	−0.866 ***	0.325	0.714 ***	0.869 ***	0.977 ***	1											
G1	−0.229 ^ns^	0.420 *	0.427 **	0.372 *	0.378 *	0.397 ^ns^	1										
G2	−0.612 ***	0.609 ***	0.972 ***	0.960 ***	0.807 ***	0.806 ***	0.444 **	1									
G3	−0.702 ***	0.634 ***	0.849 ***	0.884 ***	0.837 ***	0.885 ***	0.445 **	0.912 ***	1								
G4	−0.759 ***	0.567 ***	0.772 ***	0.863 ***	0.895 ***	0.929 ***	0.425 **	0.861 ***	0.969 ***	1							
GI	−0.669 ***	0.635 ***	0.935 ***	0.946 ***	0.846 ***	0.866 ***	0.502 **	0.982 ***	0.970 ***	0.932 ***	1						
STI_S5	−0.871 ***	0.261 ^ns^	0.696 ***	0.873 ***	0.997 ***	0.981 ***	0.357 *	0.787 ***	0.832 ***	0.896 ***	0.832 ***	1					
STI_R5	−0.879 ***	0.303 ^ns^	0.697 ***	0.858 ***	0.971 ***	0.996 ***	0.367*	0.783 ***	0.875 ***	0.926 ***	0.847 ***	0.979 ***	1				
STI_G1	0.000	0.288 ^ns^	0.075 ^ns^	0.035 ^ns^	0.007 ^ns^	0.071 ^ns^	0.750 ***	0.049 ^ns^	0.152 ^ns^	0.173 ^ns^	0.140 ^ns^	0.015 ^ns^	0.076 ^ns^	1			
STI_G2	−0.466 **	0.305 ^ns^	0.598 **	0.599 ***	0.442 **	0.482 **	0.045 ^ns^	0.548 ***	0.519 **	0.566 ***	0.542 ***	0.478 **	0.514 **	0.187 ^ns^	1		
STI_G3	−0.518 **	0.208 ^ns^	0.390 *	0.460 **	0.401 *	0.483 **	0.101 ^ns^	0.364 *	0.501 **	0.571 ***	0.434 **	0.454 **	0.528 ***	0.318 ^ns^	0.884 ***	1	
STI_G4	−0.538 ***	0.133 ^ns^	0.289 ^ns^	0.409 *	0.440 **	0.500 **	0.082 ^ns^	0.295 ^ns^	0.440 **	0.566 ***	0.373 *	0.492 **	0.547 ***	0.307 ^ns^	0.845 ***	0.970 ***	1
				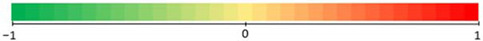													

## Data Availability

The data presented in this study are openly available.

## Supplementary Materials

The following are available online at https://www.mdpi.com/article/10.3390/plants10102137/s1, Table S1: Analysis variance of germination, morphometric parameters and drought tolerance index of shoot and root under drought stress; Table S2: Factor loadings of germination, morphological parameters and drought tolerance index under drought; Table S3: Analysis variance of germination and morphometric parameters under salt stress; Table S4: Analysis variance of salt tolerance index of germination and salt tolerance index of shoot and root (STI_R) under salt stress; Table S5: Factor loadings of germination, morphological parameters and salt tolerance index under salt stress.

## Abbreviations

Abbreviations	Full Name
HVED	high voltage electrical discharge
G	germination percentage
GI	germination index
S	shoot length
R	root length
SL_DT	shoot drought tolerance index
RL_DT	root drought tolerance index
GIDR	germination index of drought resistance
WP	control group without HVED pretreatment
PCA	principal component analysis
STI_G	salt tolerance germination index
STI_S	salt tolerance shoot index
STI_R	salt tolerance root index
