# Supplementary material for: Application of High Voltage Electrical Discharge Treatment to Improve Wheat Germination and Early Growth under Drought and Salinity Conditions
Source: Plants (Basel). 2021 Oct 9;10(10):2137. doi: 10.3390/plants10102137 (PMC8538633; doi:10.3390/plants10102137)
Supplement: Supplementary file 1 [file plants-10-02137-s001.zip › plants-1396261-supplementary.pdf]

Supplementary Materials

Table S1. Analysis variance of germination (G), germination index (GI), shoot length (S), root length (R) and drought tolerance index (SL_DTI, shoot; RL_DTI, root) under drought stress. High-voltage electrical discharge (HVED) pretreatment (P). Number beside each variable represent the day of treatment.

Source of variation	Df	G1	G2	G3	G4	GI	S2	S3	S5	R	SL_DTI	RL_DTI
**Genotype (G)**	1	18.75*	1564.1*	1220.1*	981*	334.1*	42*	221*	3132.9*	2356.2*	330.4*	500*
**Treatment (T)**	3	632.1*	1503.8*	473.4*	455.8*	1740.8*	124.9*	1054.8*	17603.3*	15912.5*	17684.4*	8143.5*
**Pretreatment (P)**	1	1064.1*	1900.1*	176.3*	93.5*	2681.6*	308*	4730.6*	1452*	2941.2*	155.4*	1036.8*
**Genotype*Treatment (G*T)**	3	17.9*	113.6*	393.1*	368.1*	145.4*	8.7*	60.1*	388.3*	863.9*	848.2*	1072.9*
**Genotype*Pretreatment (G*P)**	1	6.8*	432*	200.1*	82.7*	177.4*	4.9*	396.8*	10ns	4161.6*	178.4*	1937.9*
**Treatment*Pretreatment (T*P)**	3	160.3*	95.6*	15.2*	17.8*	102.2*	24.8*	53.7*	75.8*	182.7*	223.4*	110.7*
**Genotype*Treatment*Pretreatment (G*T*P)**	3	33.6*	88.7*	20.6*	14.1*	31.1*	5.4*	38.1*	147.6*	269.6*	129.1*	190.9*
Error	144, 108	1.1	1	1.0	1.0	1.2	0.3	1	3.5	5.6	6.5	7.2

* significant at $P \leq 0.05$; ns-not significant

32 (G1-G4, GI), 144(S2,S3,S5,R), 108(SL_DT, RL_DT)

Table S2. Factor loadings of germination, morphological parameters and drought tolerance index under drought. Germination percentage (G), germination index (GI), shoot (S) and root (R) length, drought tolerance index of shoot (SL_DT) and root (RL_DT). Number beside each variable represent the day of treatment.

Variable	PC1	PC2
**G1**	-0.80	-0.12
**G2**	-0.92	-0.25
**G3**	-0.77	-0.39
**G4**	-0.79	-0.34
**S2**	-0.82	-0.25
**S3**	-0.90	-0.22
**S5**	-0.74	0.62
**R**	-0.82	0.40
**GI**	-0.96	-0.26
**SL_DT**	-0.72	0.58
**RL_DT**	-0.84	0.40
Explained variance (eigenvalue)	7.58	1.57
Proportion of total variance (%)	68.93	14.32
Cumulative variance (%)	68.93	83.25

PC (principal component)

Table S3. Analysis variance of germination (G), shoot length (S) and root length (R) under salt stress. High-voltage electrical discharge (HVED) pretreatment (P). Number beside each variable represent the day of treatment.

Source of variation	Df	G1	G2	G3	G4	GI	S2	S3	S5	R
**Genotype (G)**	1	18.8*	10.1*	52.1*	50*	2.1*	1.2*	16*	2066.4*	573.8*
**Treatment (T)**	3	867.4*	3746.9*	4104.7*	4157.1*	4074*	329.7*	3818.3*	55523.7*	82054.4*
**Pretreatment (P)**	1	396.8*	3104.1*	3267*	2745.2*	2902.7*	60*	1651.2*	3339.8*	6262.5*
**Genotype*Treatment (G*T)**	3	21.6*	94.3*	58.1*	90.6*	11.8*	1.01*	8.9*	774.5*	328.3*
**Genotype*Pretreatment (G*P)**	1	16.3*	0.1ns	10.1*	50*	16.1*	0.4	70.2*	1.8ns	43.1*
**Treatment*Pretreatment (T*P)**	3	331.1*	644.8*	514.7*	442*	267.2*	16.7*	296.5*	501.9*	1487.4*
**Genotype*Treatment*Pretreatment (G*T*P)**	3	19.1*	16.9*	75.4*	71*	29.5*	0.3*	82.3*	24.5*	45.5*
**Error**	32, 144	0.4	1	1.2	1.1	0.8	0.1	0.2	1.0	8.6

* significant at $P \le 0.05$; ns-not significant

32(G1-G4), 144(S2, S3, S4, R)

Table S4. Analysis variance of salt tolerance index of germination during four days (STI_G), salt tolerance index of shoot (STI_S) and root (STI_R) under salt stress. High-voltage electrical discharge (HVED) pre-treatment (P). Number beside each variable represent the day of treatment.

Source of variation	Df	STI_G1	STI_G2	STI_G3	STI_G4	STI_S2	STI_S3	STI_S5	STI_R
**Genotype (G)**	1	0.00*	0.06*	0.00*	0.00*	0.01*	0.00*	0.12*	0.02*
**Treatment (T)**	2	0.00*	0.84*	1.16*	1.46*	0.54*	1.76*	2.18*	2.25*
**Pretreatment (P)**	1	0.00*	1.34*	1.79*	1.54*	1.23*	0.43*	0.33*	0.57*
**Genotype*Treatment (G*T)**	2	0.00*	0.04*	0.04*	0.07*	0.00*	0.02*	0.11*	0.03*
**Genotype*Pretreatment (G*P)**	1	0.00*	0.00ns	0.00*	0.03*	0.01*	0.08*	0.00*	0.00*
**Treatment*Pretreatment (T*P)**	2	0.00*	0.38*	0.13*	0.11*	0.54*	0.10*	0.09*	0.17*
**Genotype*Treatment*Pretreatment (G*T*P)**	2	0.00*	0.01*	0.05*	0.04*	0.01*	0.05*	0.00*	0.00ns
**Error**	24, 108	0.00	0.00	0.00	0.00	0.00	0.00	0.00	0.00

* significant at $P \leq 0.05$; ns-not significant

24 (STI_G1- STI_G4), 108 (STI_S2, STI_S3, STI_S5, STI_R)

Table S5. Factor loadings of germination, morphological parameters and salt tolerance index under salt stress. Germination percentage (G), germination index (GI), shoot (S) and root (R) length, salt tolerance index of shoot (STI_S), root (STI_R) and germination (STI_G). Number beside each variable represent the day of treatment.

Variable	PC1	PC2
G1	-0.870	-0.456
G2	-0.966	-0.219
G3	-0.948	0.100
G4	-0.938	0.262
GI	-0.980	-0.063
S2	-0.906	-0.405
S3	-0.984	-0.111
S5	-0.915	0.306
R	-0.938	0.322
STI_S2	-0.926	-0.334
STI_S3	-0.943	0.105
STI_S5	-0.907	0.335
STI_R5	-0.929	0.340
STI_G1	-0.842	-0.477
STI_G2	-0.971	-0.156
STI_G3	-0.951	0.115
STI_G4	-0.939	0.274
Explained variance (eigenvalue)	14.808	1.403
Proportion of total variance (%)	87.105	8.253
Cumulative variance (%)	87.105	95.358

PC1 (principal component 1); PC2 (principal component 2)
